# Electronic health records and patient registries in medical oncology departments in Spain

**DOI:** 10.1007/s12094-021-02614-9

**Published:** 2021-04-17

**Authors:** N. Ribelles, I. Alvarez-Lopez, A. Arcusa, J. I. Chacon, J. de la Haba, J. García-Corbacho, J. Garcia-Mata, C. Jara, J. M. Jerez, M. Lázaro-Quintela, L. Leon-Mateos, N. Ramirez-Merino, A. Tibau, A. Garcia-Palomo

**Affiliations:** 1 Medical Oncology Intercenter Unit, Regional and Virgen de la Victoria University Hospitals, IBIMA, Hospital Universitario Virgen de la Victoria, Campus Teatinos s/n., 29010 Málaga, Spain; 2 Medical Oncology Department, Hospital Universitario Donostia-BioDonostia, Donostia, Gipuzkoa Spain; 3 Oncology Department, Consorci Sanitari de Terrassa, Barcelona, Spain; 4 Medical Oncology Department, Hospital Virgen de la Salud, Toledo, Spain; 5 Medical Oncology Department, Hospital Universitario Reina Sofía, Instituto de Investigación Biomédica de Córdoba (IMIBIC), Córdoba, Spain; 6 Medical Oncology Department, Hospital Clinic/IDIBAPs, Barcelona, Spain; 7 Medical Oncology Department, Complexo Hospitalario Universitario de Ourense, Ourense, Spain; 8 Oncology Department, Hospital Universitario Fundación Alcorcón, Madrid, Spain; 9 Department of Languages and Computer Science, E.T.S.I. Ingenería Informática, University of Málaga, Málaga, Spain; 10 Medical Oncology Department, Hospital Álvaro Cunqueiro, Vigo, Spain; 11 Medical Oncology Department, Complexo Hospitalario Universitario de Santiago de Compostela, Health Research Institute of Santiago (IDIS), Santiago de Compostela, Spain; 12 Oncología Médica, Hospital Universitario Infanta Elena, Madrid, Spain; 13 Medical Oncology Department, Hospital de la Santa Creu i Sant Pau, Barcelona, Spain; 14 Medical Oncology Department, Complejo Asistencial Universitario de León, Instituto de Biomedicina (IBIOMED), León, Spain

**Keywords:** Electronic health records, Patient registries, Medical oncology, Work organization, Clinical practice, Clinical research

## Abstract

**Purpose:**

We aimed to evaluate the current situation of electronic health records (EHRs) and patient registries in the oncology departments of hospitals in Spain.

**Methods:**

This was a cross-sectional study conducted from December 2018 to September 2019. The survey was designed ad hoc by the Outcomes Evaluation and Clinical Practice Section of the Spanish Society of Medical Oncology (SEOM) and was distributed to all head of medical oncology department members of SEOM.

**Results:**

We invited 148 heads of oncology departments, and 81 (54.7%) questionnaires were completed, with representation from all 17 Spanish autonomous communities. Seventy-seven (95%) of the respondents had EHRs implemented at their hospitals; of them, over 80% considered EHRs to have a positive impact on work organization and clinical practice, and 73% considered that EHRs improve the quality of patient care. In contrast, 27 (35.1%) of these respondents felt that EHRs worsened the physician–patient relationship and conveyed an additional workload (*n* = 29; 37.6%). Several drawbacks in the implementation of EHRs were identified, including the limited inclusion of information on both outpatients and inpatients, information recorded in free text data fields, and the availability of specific informed consent. Forty-six (56.7%) respondents had patient registries where they recorded information from all patients seen in the department.

**Conclusion:**

Our study indicates that EHRs are almost universally implemented in the hospitals surveyed and are considered to have a positive impact on work organization and clinical practice. However, EHRs currently have several drawbacks that limit their use for investigational purposes.

**Clinical trial registration:**

Not applicable

**Supplementary Information:**

The online version contains supplementary material available at 10.1007/s12094-021-02614-9.

## Introduction

According to the International Organization for Standardization (ISO) [1], an electronic health record (EHR) is “a repository of patient data in digital form, stored and exchanged securely, and accessible by multiple authorized users. It contains retrospective, concurrent, and prospective information, and its primary purpose is to support continuing, efficient and quality integrated health care”. A previous systematic review has shown that EHRs improve the quality of health care by reducing documentation time, increasing guideline adherence, and reducing medication errors and drug-related adverse events [2]. Although the implementation of EHRs has not been shown to reduce mortality [2, 3], some studies have demonstrated that they can moderately improve morbidity outcomes [3].

EHRs may also be an important tool for clinical research. Within the oncology field, they have been used for evaluating the cancer risk associated with some treatments [4, 5] and to create predictive models [6, 7] that allow personalized care and to evaluate the quality of cancer care [8], to cite some examples. In clinical trials, EHRs facilitate accessibility to medical records and thus speed up the identification of potential participants [9, 10]. They also facilitate the capture of electronic data in an automated way [11], helping to monitor the review of source data, such as EHR, and thus avoid difficulties in interpreting the handwritten characters of doctors. In addition, the availability of electronic tools to find specific words in long text and even potentially allow remote monitoring, which recently became a major issue during the pandemic, is useful. Additionally, EHRs can be a source for selecting patients treated with a contemporary standard of care as an external control to interpret the efficacy observed in early phase single-group clinical trials [12]. Early in drug development, EHRs may be used to identify potential candidates for the treatment of cancer by analyzing the effects on survival of noncancer drugs in patients with cancer—so-called drug repurposing [13, 14]. EHRs are also an important source for obtaining real-world evidence for the effects of interventions [15]. In this regard, the American Society of Clinical Oncology in collaboration with the MITRE Corporation (a private, not-for-profit company providing engineering and technical guidance for the federal government in the US) launched an initiative in 2019, the mCODE (Minimal Common Oncology Data Elements), for establishing a core set of structured data elements for oncology EHRs that would enable the treatment of every cancer patient to contribute to comparative effectiveness analysis [16, 17].

There is no information on the availability and characteristics of EHRs in oncology at the country level in Spain. Therefore, the Outcomes Evaluation and Clinical Practice Section of the Spanish Society of Medical Oncology (SEOM) conducted this survey with the aim of evaluating the degree of implementation of EHRs by the medical oncology departments of hospitals in Spain, describing the situation regarding cancer registries among those departments, and assessing their willingness to share patient data.

## Subjects and methods

### Study design

This was a cross-sectional study that was undertaken from December 2018 to September 2019. Since the survey was addressed to health professionals and no individual patient datum was recorded, evaluation by the ethics committee was not required. Participants were informed of the objectives of the survey and that their participation was not completely anonymous. That is, centers were identified in the questionnaire, but the health professional reporting the information was not. They were also informed that the results of the survey would be reported in an aggregate manner.

### The survey

The survey was designed ad hoc by 11 members (NR, AGP, IAL, AAL, JGM, JHR, CJS, MLQ, LLM, NRM and ATM) of the Outcomes Evaluation and Clinical Practice Section of the SEOM, which in turn supported this study. The survey was distributed by email to all SEOM members with the category of head of medical oncology department with a cover letter explaining the objectives of the study. Three reminders were sent via email. Finally, the members of the Executive Committee of this section of SEOM tried to contact the nonresponders directly via email or phone.

The surveyed comprised 17 questions that could be grouped into the following sections:

1. Practice identification and age, position and experience of the health professional who filled out the questionnaire (1 question).
2. Data on the availability of patient registries in the department, type of registries, and need for a national cancer registry (3 questions).
3. Data on the availability of an electronic prescription system and its characteristics (2 questions).
4. Data on EHRs (11 questions):
  - EHR availability
  - Length of time that EHR has been available
  - It is considered of interest in implementing EHR in the department if not available
  - What are the characteristics of the information included in EHRs?
  - In which format the data are recorded
  - Degree of agreement on the impact of the availability of an EHR on several issues (measures on a 3-point Likert scale)
  - Availability of tools for extracting data from EHR
  - Possibility of accessing diagnostic tests through the EHR
  - Compatibility of the EHR for use in clinical trials
  - Availability of informed consent for using patient information from the EHR
  - Willingness to share information from the EHRs with other investigators.

The complete questionnaire is available as Supplementary Information, both in the English and Spanish versions.

### Statistical analysis

We used a convenience sample; therefore, we did not perform any sample size calculations.

Our data are essentially descriptive. We used absolute and relative frequencies to present qualitative variables.

All analyses were performed using Microsoft Excel 365.

## Results

### Response rate and characteristics of the respondents

We invited 148 heads of oncology departments throughout Spain, and after reminders, 81 (54.7%) answered the questionnaires. All 17 Spanish autonomous communities (AACC) and 1 of the 2 Spanish autonomous cities were represented by at least one hospital. However, hospitals from 5 AACCs represented 64.1% of the responses: Catalonia (*n* = 14, 17.5%), Madrid (*n* = 11, 13.6%), Andalusia (*n* = 11, 13.6%), Valencia (*n* = 9, 11.1%), and Galicia (*n* = 7, 8.6%) (Table 1). Questionnaires were completed by the head of the department (*n* = 54, 66.6%), followed by the head of section (*n* = 21, 25.9%) and the senior registrar (*n* = 6, 7.4%). Seventy-three (90.1%) of the respondents were older than 45 years, and 66 (81.4%) had professional experience greater than 20 years.

**Table 1:** Number of hospitals with answered surveys

Spanish autonomous communities	*N*
Andalusia	11 (13.5%)
Aragon	3 (3.7%)
Asturias	1 (1.2%)
Balearic Islands	2 (2.4%)
Basque Country	3 (3.7%)
Canary Islands	1 (1.2%)
Cantabria	1 (1.2%)
Castile and Lion	4 (4.9%)
Castilla La Mancha	4 (4.9%)
Catalonia	14 (17.3%)
Ceuta	1 (1.2%)
Estremadura	3 (3.7%)
Galicia	7 (8.6%)
Madrid	11 (13.5%)
Murcia	3 (3.7%)
Navarre	1 (1.2%)
Rioja	2 (2.4%)
Valencia	9 (11.1%)

### Availability and characteristics of patient registries

**Fig. 1:**
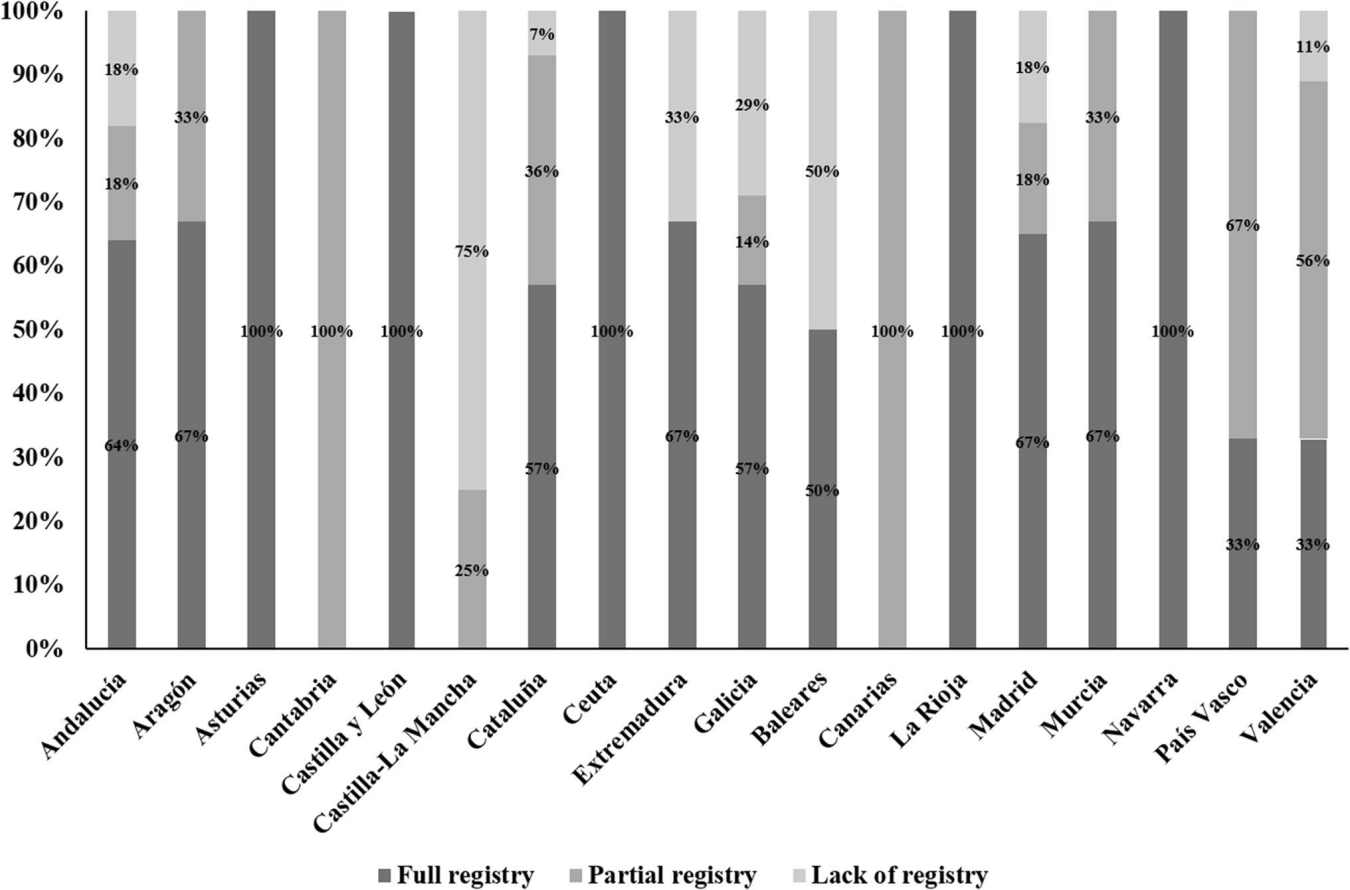
Availability of patient registries by autonomous communities

Forty-six (56.7%) of the centers had a register or database where the information of all patients attending the clinical offices was recorded. There were no relevant differences in this regard among the AACCs with the largest number of participant centers, except for Valencia, who reported that only 3 out of the 9 (33.3%) participant hospitals had a patient registry/database (Fig. 1). There were only five AACCs in which 100% of the responders registered all patients treated in their services (Asturias, Castilla-León, Ceuta, La Rioja and Navarra). Additionally, 22 (27.1%) respondents reported the presence of other registries conducted by some of the medical staff of the department. The information recorded in a patient registry/database was the type of cancer (*n* = 73, 90.1%), stage (*n* = 55, 67.9%), date of diagnosis (*n* = 51, 62.9%), type of treatment received (*n* = 46, 56.7%) and date and clinical status at the last follow-up visit (*n* = 27, 33.3%).

All but 2 of the respondents (97.5%) considered that there was a need to create a national registry of cancer patients.

### Availability of an electronic prescription system and its characteristics

Seventy-six out of the 81 participants (93.8%) reported having an electronic prescription system. Of those with the electronic prescription system, 42 (55.2%) had direct access to the electronic prescription system from the EHR, 33 (43.4%) could directly obtain information about the prescribed treatments, 30 (39.4%) could request that information through the Pharmacy Department and 3 (3.9%) could not obtain that information.

### Electronic health record

Seventy-seven (95.1%) of the respondents had EHRs implemented at their hospitals, and of them, 63 (81.8%) had used that tool for a period longer than 5 years. All four respondents without an EHR implemented at their center considered that, currently, the EHR is an essential tool.

**Fig. 2:**
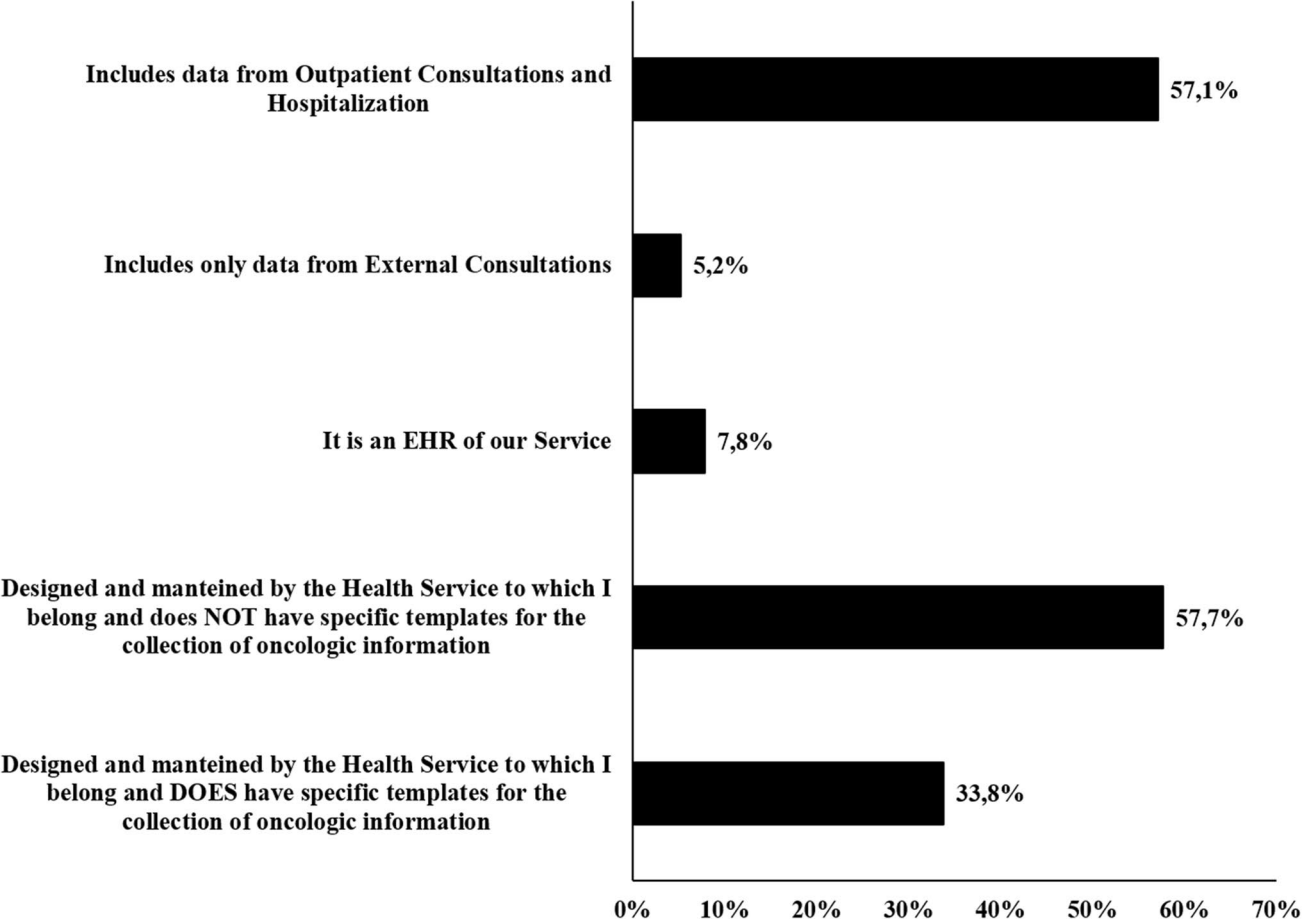
Characteristics of the electronic health record

In over 90.9% of cases, the EHR was designed and maintained by the AACC Health Service, but only one-third had specific templates for recording information (Fig. 2). In 57.1% of the centers, the EHR includes information on outpatient consultation, as well as on hospitalized patients. Data from the EHR are recorded in a structured manner (i.e., using drop-down lists) in 10.4% of the centers, while 48.1% of them use free text data fields, and 41.5% combine the two systems.

**Fig. 3:**
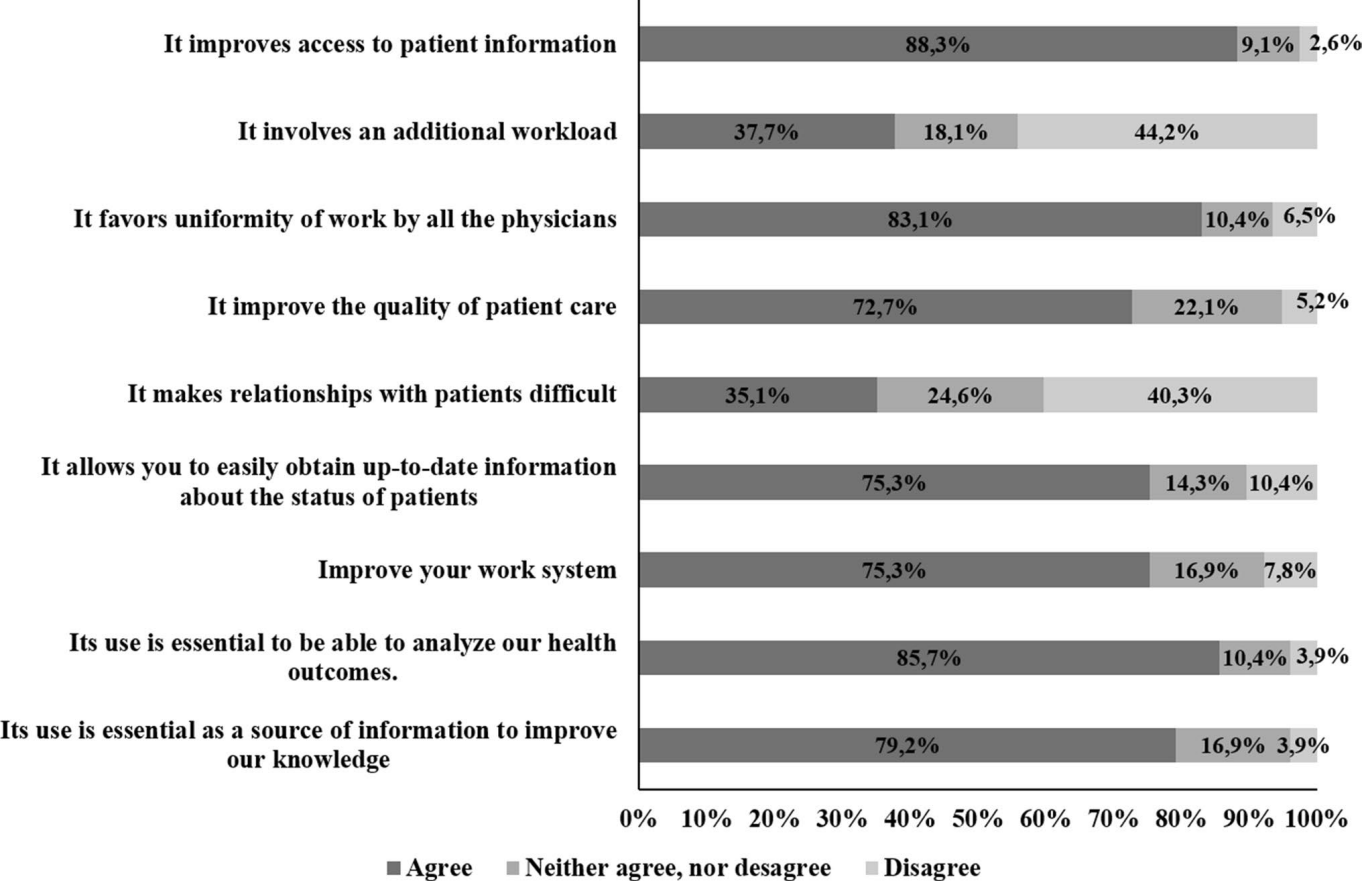
Impact of the electronic health record

In the survey, several questions were included to determine opinions about different aspects related to the use of EHRs. More than eighty percent considered that its use improves access to patient information (68; 88.3%), that it is essential to analyze health outcomes (66; 85.7%) and that it favors uniformity of work by all physicians (64; 83.1%). Between seventy and eighty percent supported the value of EHR as an essential source of information to improve knowledge (61; 79.2%) and to progress in our work system (58; 75.3%). In addition, the use of EHRs allows us to easily obtain updated information about patient status (58; 75.3%) and contributes to improving the quality of patient care (56: 72.7%). However, there was no such clear agreement on whether use of the EHR worsened the physician–patient relationship (yes 27; 35.1% vs no 31; 40.2%) or whether it involved an additional workload (yes 29; 37.6% vs no 34; 44.1%) (Fig. 3).

**Fig. 4:**
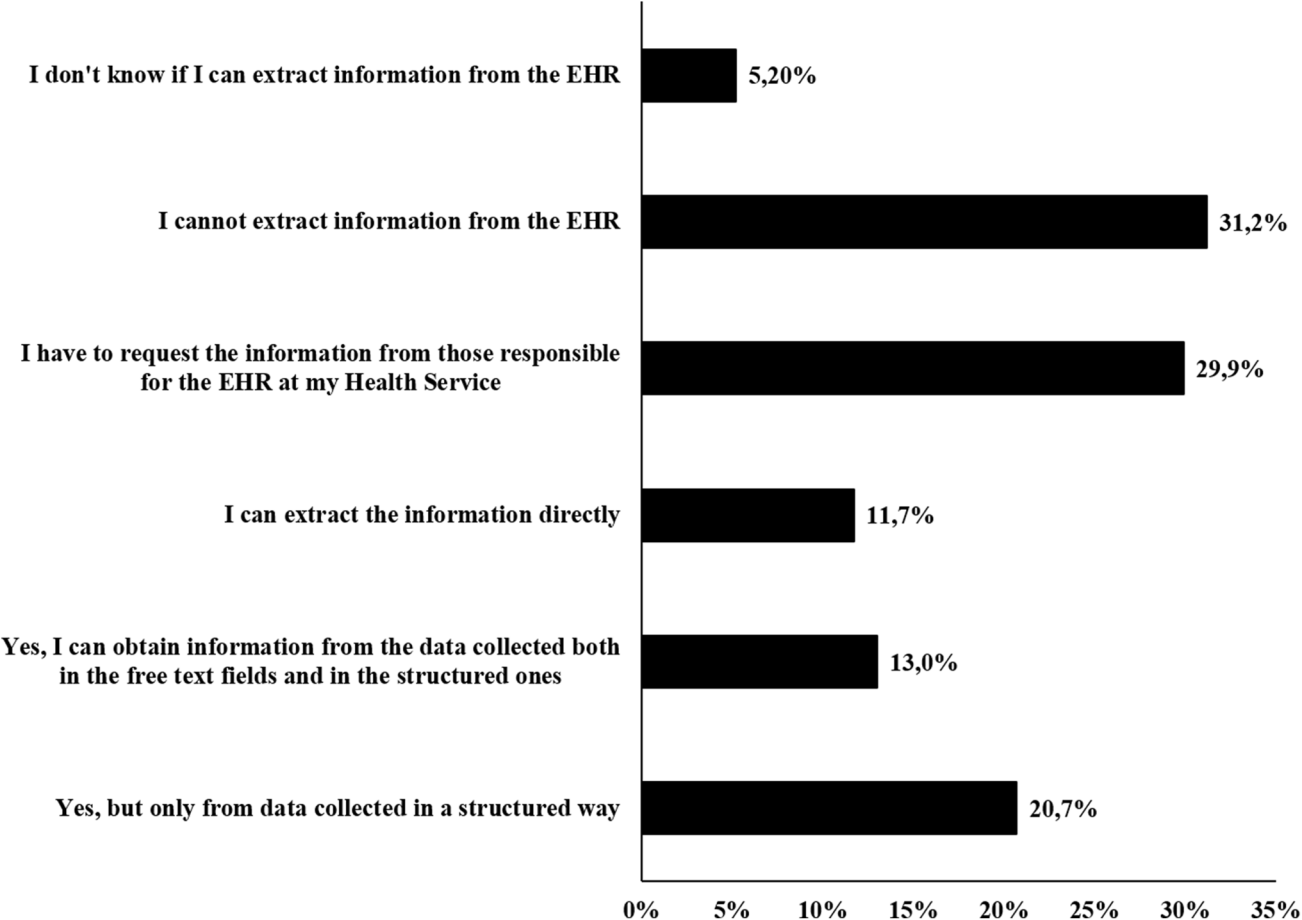
Information extraction from the electronic health record

Regarding extraction of data from the EHRs (Fig. 4), 24 (31.2%) respondents reported that they could not extract information. Only 9 (11.7%) of the respondents could directly extract information from the EHRs, and 23 (29.8%) had to request the information from those responsible for the EHRs within the AACC Health Service. Only 32 (41.5%) respondents could directly access information from diagnostic tests from the EHR. Forty-eight (62.3%) respondents thought that their EHR met the needed features to be considered a proper EHR by the sponsors of clinical trials. Thirty-five respondents (45.4%) reported that their patients signed a general informed consent form for the administration of treatments in which the use of their data collected in the EHR was also authorized. Moreover, sixteen other respondents (20.8%) pointed out that their patients signed a specific consent form to authorize the use of their data.

**Fig. 5:**
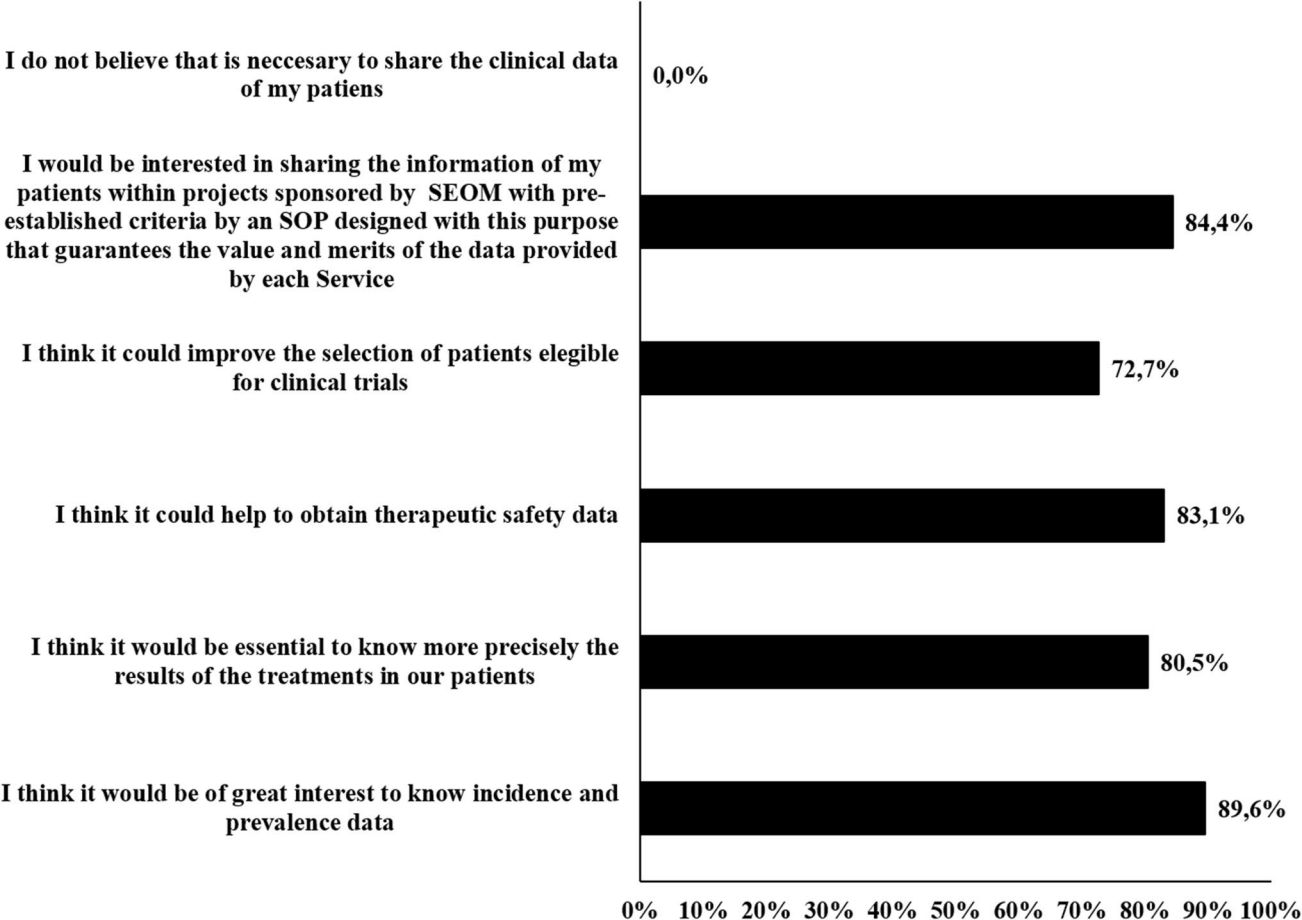
Opinion on sharing patient data from EHRs

Overall, nearly 85% of the respondents were willing to share data from EHRs within the frame of a project sponsored by SEOM and considered that sharing data might have a positive impact on obtaining information on health outcomes from cancer patients and would allow one to obtain information on the prevalence and incidence of cancer (Fig. 5).

## Discussion

Our study indicates that EHRs are almost universally implemented in the hospitals surveyed and are considered to have a positive impact on work organization and clinical practice. However, there are several drawbacks to how EHRs are implemented that limit optimal use for both clinical practice and clinical research.

According to our survey, 95.1% of the participating hospitals had implemented an EHR, a figure that is entirely consistent with the 93.3% coverage of the National Health System Electronic Health Records Project reported by the Spanish Ministry of Health as stated in the last report of October 2020 [18]. Those hospitals where an EHR is not yet available considered it an essential tool. Most respondents believe that the availability of EHRs has a positive impact on work organization and clinical practice and agree that it improves the quality of patient care. However, it is important to note that 35.1% of surveyed oncologists think that the EHR may interfere with the doctor–patient relationship. This concern is somehow justified, as the use of EHRs may disrupt communication (e.g., interrupted speech), and this perception of a negative impact on the patient–doctor relationship has been reported previously [19]. However, a systematic review found that EHRs produce no change in patient satisfaction or patient–doctor communication [20]. Our study also shows that 37.6% of the centers surveyed consider that EHRs increase workload. Studies conducted in primary care show that, in fact, the implementation of EHRs can improve productivity/efficiency in physician workloads [21]. Improving the usability of the EHR may decrease this perception of increased workload [22].

Although two-thirds of respondents reported that their EHRs meet the needed characteristics for being considered a proper EHR by sponsors of clinical trials, in our view, there are still many drawbacks in the implementation of EHRs in Spain that limit their use for research purposes, including clinical trials. Only 57.1% of centers have an EHR that includes information on both outpatients and inpatients. Almost half of centers use an EHR where the information is recorded in free text data fields. Approximately two-thirds of respondents either cannot extract information from the EHR or must request the desired information from the person responsible for this system at the AACC Health System.

We did not include questions in our survey regarding the specific content of the EHR. The ASCO project mCODE recommends core elements be included for cancer research treatment, including patient information (demographics, comorbid conditions and performance status), cancer characteristics, genomics information, laboratory and vital sign information, including tumor markers, cancer treatment information, and cancer disease status [16]. We think this information could only be obtained in our setting by designing and implementing the appropriate tools that allow us to extract and analyze this information from the content of EHRs. Fortunately, over 80% of respondents were willing to share their data, but this could be hampered by the fact that it is not possible to share the data contained in the EHRs from the health systems from different AACCs. Therefore, by involving additional stakeholders (i.e., pharmacists, hospital management, and scientific societies, such as SEOM, among others), similar projects could be implemented in Spain in the future.

Nearly 84% of the centers surveyed had some type of patient register or database, and the registry included information on all patients attended in 56.7% of the surveyed hospitals. It is possible that this high number of centers with registries was facilitated by the high use of EHRs. In the United States, it has been reported that the availability of EHRs facilitates the development and implementation of patient registries [23]. The information included in the registries from our survey is limited, especially for evaluating treatment outcomes, since only 56.7% recorded the type of treatment received, and only 33.3% recorded the clinical status at the last follow-up. This situation is further complicated by the fact that despite the availability of an electronic prescription system in 93.8% of centers, 43.3% could not directly obtain information on prescribed treatments.

A limitation of our survey is the relatively low rate of participants, since only 54.7% of the hospitals that were contacted answered the questions proposed. Therefore, despite the involvement of centers from all except one administrative territory of Spain, our results cannot be considered representative of the situation at a national level. Nevertheless, the high percentages of agreement obtained in certain responses supports the representativeness of our results. It is also possible that the participants in our survey were those more interested in the use of EHRs, and their opinion could be biased to a positive perception of the usefulness of EHRs. However, the importance of EHRs in oncology research is indubitable [23, 24], especially with the increasing relevance of real-world data [25].

According to data obtained by our national survey, the implementation and use of EHRs in Spain appears to have a wide and extensive distribution. However, the EHR's reliance on seventeen different healthcare systems results in several notable weaknesses. Information systems are not compatible with each other and do not always collect the same data. Therefore, it is not possible in any way to obtain information for joint analysis of large-scale data or to compare health outcomes between hospitals or autonomous communities. Furthermore, in most EHRs, it is not possible to obtain or analyze valuable health outcomes.

On the other hand, the use of patient registries is still less implemented. Not all medical oncology departments have registries of the patients they attend, and if they do, they do not always include all their patients or collect enough data. In this sense, it should be highlighted that practically all of the oncologists surveyed consider it necessary to create a National Cancer Registry like those available in most countries of the same level.

The development and implementation of a National Cancer Registry first and a national EHR in the second should be an objective of the health authorities, using consensus as a way to establish what collected data is considered essential. Of course, a national oncology information system of these characteristics should have the appropriate tools to extract data and health outcomes. There is no doubt that explicit support and legal coverage from health authorities are essential for the implementation of such an information system. However, the technology necessary for its development could come from other collaborative sources of the academic and university environment.

Finally, it cannot be ignored that analysis of the data collected in an information system of such characteristics would allow planning the distribution of available resources for the care of cancer patients in the most efficient way.

## Supplementary Information

Below is the link to the electronic supplementary material.

Supplementary file1 (DOCX 19 KB)

Supplementary file2 (DOCX 19 KB)
