# Supplementary material for: Electronic health records and patient registries in medical oncology departments in Spain
Source: Clin Transl Oncol. 2021 Apr 17;23(10):2099–108. doi: 10.1007/s12094-021-02614-9 (PMC8390424; doi:10.1007/s12094-021-02614-9)
Supplement: Supplementary file 1 — Supplementary file1 (DOCX 19 KB) [file 12094_2021_2614_MOESM1_ESM.docx]

**Electronic health records and patient registries in medical oncology departments in Spain**

**Nuria Ribelles^1^, Isabel Alvarez-Lopez^2^, Angels Arcusa^3^, Jose Ignacio Chacon^4^, Juan de la Haba^5^, Javier García-Corbacho^6^, Jesus Garcia-Mata^7^, Carlos Jara^8^, José Manuel Jerez^9^, Martín Lázaro-Quintela^10^, Luis Leon-Mateos^11^, Natalia Ramirez-Merino^12^, Ariadna Tibau^13^, Andrés Garcia-Palomo^14^**

**SUPPLEMENTARY INFORMATION**

**Survey questionnaire**

1. **In your Service, is there a database or registry of patients?**
   1. Yes, all patients arriving at the Service are registered.
   2. Yes, some doctors register their patients.
   3. We do not have any type of registry or database.
2. **In the records that you have in your Service, information is collected about (indicate all the options you consider):**
   1. Type of neoplasm.
   2. Stage.
   3. Type of treatment performed.
   4. Diagnosis date.
   5. Date and status of last control.
   6. Others.
3. **Do you think it is necessary to create a National Registry of cancer patients?**
   1. Yes.
   2. No.
4. **Do you have an application or electronic prescription system?**
   1. Yes.
   2. No.
5. **If you have an electronic prescription system (indicate all the options you consider):**
   1. I can access it directly from my EHR.
   2. It is not part of the EHR and I have to access another application to use it.
   3. I can do both in-hospital and out-of-hospital prescriptions.
   4. I can obtain information directly from the data collected about the prescribed treatments.
   5. I can get information, but I have to request it from the Pharmacy Service.
   6. I cannot get information about the prescribed treatments.
   7. I don't know if I can get information about the prescribed treatments.
6. **Do you have an Electronic Medical Record (EHR)?**
   1. No (Continue with question 7 and finish the questionnaire).
   2. Yes (Continue from question 8).
7. **If you do not have an EHR, would you be interested in being able to use a tool of these characteristics in your clinical practice?**
   1. Yes, I think they are currently essential.
   2. Yes, although I think it would mean an additional workload but it would be offset by their profits.
   3. No, I think it would slow down my work and not bring me any benefit.
8. **How long have you been using EHR?**
   1. Less than 2 years.
   2. 2-5 years.
   3. > 5 years.
9. **The EHR you use (check all that you consider):**
   1. It is designed and maintained by the Health System to which I belong and DOES have specific templates for the collection of oncological information.
   2. It is designed and maintained by the Health System to which I belong and does NOT have specific templates for the collection of oncological information.
   3. It is an EHR of our Service.
   4. Includes only data from Outpatient Consultations.
   5. Includes only Hospitalization data.
   6. Includes data from Outpatient Consultations and Hospitalization.
10. **The information in your EHR:**
    1. It is collected in a structured way (drop-down menus).
    2. It is collected in free text fields.
    3. Both.
11. **Regarding the EHR:**

|  | **IN**  **AGREEMENT** | **NEITHER AGREE NOR DISAGREE** | **IN DISAGREEMENT** |
| --- | --- | --- | --- |
| Improve access to patient information |  |  |  |
| It involves an additional workload |  |  |  |
| Encourages all physicians work in a more uniform manner |  |  |  |
| Improve the quality of patient care |  |  |  |
| Difficulty relationships with patients |  |  |  |
| It allows you to easily obtain up-to-date information about the status of patients |  |  |  |
| Improve your work system |  |  |  |
| Its use is essential to be able to analyze our health outcomes. |  |  |  |
| Its use is essential as a source of information to improve our knowledge. |  |  |  |

1. **Do you have any application or tool to extract information from your EHR? (check all the options you consider):**
   1. Yes, but only from data collected in a structured way.
   2. Yes, I can get information from the data collected in both free text and structured fields.
   3. I can extract the information directly.
   4. I have to request the information from those responsible for the EHR at my Health System.
   5. I cannot extract information from the EHR.
   6. I don't know if I can extract information from the EHR.
2. **Regarding requests and results of diagnostic tests:**
   1. I can access them directly from my EHR and incorporate the results.
   2. I can access them directly but if I want to incorporate the results I have to make a cut-paste.
   3. To access them I have to open other applications.
3. **Does your EHR meet the necessary characteristics to be considered as such by the clinical trials promoters?:**
   1. Yes.
   2. No.
   3. I do not know.
4. **Regarding the Informed Consent of patients to access their data:**
   1. They sign a general consent for the application of treatments or upon arrival at the Service, which also states that we can use the data contained in the EHR.
   2. They sign a specific consent to use their medical records.
   3. I do not know if any of the consents they sign refers to the use of their clinical data.
5. **Regarding sharing the information of your patients collected in your EHR (check all the options you consider):**
   1. I think it would be of great interest to know incidence and prevalence data.
   2. I think it would be essential to know more precisely the results of the treatments in our patients.
   3. I think it could help to obtain therapeutic safety data.
   4. I think it could improve the selection of patients eligible for clinical trials.
   5. I would be interested in sharing the information of my patients within projects sponsored by the SEOM with pre-established criteria by an SOP designed for this purpose that guarantees the value and merits of the data provided by each Service.
   6. I do not believe that it is necessary to share the clinical data of my patients.
