# Supplementary material for: Electronic health records and patient registries in medical oncology departments in Spain
Source: Clin Transl Oncol. 2021 Apr 17;23(10):2099–108. doi: 10.1007/s12094-021-02614-9 (PMC8390424; doi:10.1007/s12094-021-02614-9)
Supplement: Supplementary file 2 — Supplementary file2 (DOCX 19 KB) [file 12094_2021_2614_MOESM2_ESM.docx]

**CUESTIONARIO DE LA ENCUESTA.**

1. **Datos identificación.**
   1. Nombre del Hospital.
   2. Comunidad Autónoma (Andalucía, Aragón, Asturias, Canarias, Castilla y León, Castilla-La Mancha, Cataluña, Comunidad Valenciana, Galicia, Islas Baleares, Madrid, Murcia, País Vasco)
   3. Tipo de práctica clínica (Pública, Privada, Ambas).
   4. Puesto que ocupa en el Servicio (Jefe de Servicio, Jefe de Sección, Adjunto, Residente).
   5. Edad (25-35, 36-45, 46-55, >55).
   6. Años de práctica (<5, 5-10, 10-20, >20).
2. **En tu Servicio, ¿se dispone de alguna base de datos o registro de pacientes?**
   1. Sí, se registran todos los pacientes que llegan al Servicio.
   2. Sí, algunos facultativos registramos los pacientes que visitamos en la consulta.
   3. No disponemos de ningún tipo de registro o base de datos.
3. **En el/los registros que tenéis en** **vuestro Servicio** **se recoge información acerca de (señala todas las opciones que consideres):**
   1. Tipo de neoplasia.
   2. Estadio.
   3. Tipo de tratamiento realizado.
   4. Fecha diagnóstico.
   5. Fecha y estado de ultimo control.
   6. Otros
4. **¿Consideras que es necesario crear un Registro Nacional de pacientes con cáncer?**
   1. Si.
   2. No.
5. **¿Dispones de algún aplicación o sistema de prescripción electrónica?**
   1. Sí.
   2. No.
6. **Si dispones de un sistema de prescripción electrónica (señala todas las opciones que consideres):**
   1. Puedo acceder a él directamente desde mi HCE.
   2. No forma parte de la HCE y tengo que acceder a otra aplicación para usarlo.
   3. Puedo realizar prescripciones tanto intrahospitalarias como extrahospitalarias.
   4. Puedo obtener información directamente de los datos recogidos acerca de los tratamientos prescritos.
   5. Puedo obtener información, pero tengo que solicitarla al Servicio de Farmacia o a los SSCC.
   6. No puedo obtener información acerca de los tratamientos prescritos.
   7. Desconozco si puedo obtener información acerca de los tratamientos prescritos.
7. **¿Dispones de Historia Clínica Electrónica (HCE)?**
8. No (Continuar con la pregunta 8 y finalizar el cuestionario).
9. Si (Continuar a partir de la pregunta 9).
10. **Si no dispones de HCE, ¿estarías interesado en poder utilizar una herramienta de estas características en tu práctica clínica?**
    1. Sí, creo que hoy en día son imprescindibles.
    2. Sí, aunque creo que supondría una carga de trabajo adicional pero quedaría compensado por sus utilidades.
    3. No, creo que enlentecería mi trabajo y no me aportaría ningún beneficio.
11. **¿Desde hace cuánto tiempo usas HCE?**
    1. Menos de 2 años.
    2. 2-5 años.
    3. >5 años.
12. **La HCE que utilizas (señala todas las que consideres):**
    1. Está diseñada y mantenida por el Servicio de Salud al que pertenezco y SI dispone de plantillas específicas para la recogida de la información oncológica.
    2. Está diseñada y mantenida por el Servicio de Salud al que pertenezco y NO dispone de plantillas específicas para la recogida de la información oncológica.
    3. Es una HCE propia de nuestro Servicio.
    4. Incluye solo datos de Consultas Externas.
    5. Incluye solo datos de Hospitalización.
    6. Incluye datos de Consultas Externas y de Hospitalización.
13. **La información en tu HCE:**
    1. Se recoge de manera estructurada (menús desplegables).
    2. Se recoge en campos de texto libre.
    3. Ambas.
14. **Con respecto a la HCE:**

|  | **DE ACUERDO** | **NI DE ACUERDO NI EN DESACUERDO** | **EN DESACUERDO** |
| --- | --- | --- | --- |
| Mejora el acceso a la información de los pacientes |  |  |  |
| Supone una carga de trabajo adicional. |  |  |  |
| Favorece que todos los facultativos trabajen de una manera más uniforme. |  |  |  |
| Mejora la calidad en la asistencia a los pacientes. |  |  |  |
| Dificulta la relación con los pacientes. |  |  |  |
| Permite obtener fácilmente información actualizada acerca del estado de los pacientes. |  |  |  |
| Mejora tu sistemática de trabajo. |  |  |  |
| Su uso es imprescindible para poder analizar nuestros resultados en salud. |  |  |  |
| Su uso es imprescindible como fuente de información para mejorar nuestro conocimiento. |  |  |  |

1. **¿Dispones de alguna aplicación o herramienta para extraer información de tu HCE? (señala todas las opciones que consideres):**
   1. Sí, pero solo de los datos recogidos de manera estructurada.
   2. Sí, puedo obtener información de los datos recogidos tanto en los campos de texto libre como en los estructurados.
   3. Puedo extraer la información directamente.
   4. Tengo que solicitar la información a los responsables de la HCE a mi Servicio de Salud.
   5. No puedo extraer información de la HCE.
   6. Desconozco si puedo extraer información de la HCE.
2. **Con respecto a las solicitudes y resultados de exploraciones diagnósticas:**
   1. Puedo acceder a ellas directamente desde mi HCE e incorporar los resultados.
   2. Puedo acceder directamente a ellas pero si quiero incorporar los resultados tengo que hacer un corta-pega.
   3. Para acceder a ellas tengo que abrir otras aplicaciones.
3. **Tu HCE ¿reúne las características necesarias para ser considerada como tal por los promotores de EECC?:**
   1. Sí.
   2. No.
   3. Lo desconozco.
4. **Con respecto al Consentimiento Informado de los pacientes para poder acceder a sus datos:**
   1. Firman un consentimiento general para la aplicación de tratamientos o a su llegada al Servicio, en el que también consta que podemos utilizar los datos contenidos en la HCE.
   2. Firman un consentimiento específico para poder utilizar su historia clínica.
   3. Desconozco si en cualquiera de los consentimientos que firman se hace referencia al uso de sus datos clínicos.
5. **Con respecto a compartir la información de tus pacientes recogida en tu HCE (señala todas las opciones que consideres):**
   1. Creo que resultaría de gran interés para conocer datos de incidencia y prevalencia.
   2. Creo que sería imprescindible para conocer con mayor precisión los resultados de los tratamientos en nuestros pacientes.
   3. Creo que podría ayudar a obtener datos de seguridad terapéutica.
   4. Creo que podría mejorar la selección de pacientes elegibles para EECC.
   5. Estaría interesado en compartir la información de mis pacientes dentro de proyectos auspiciados por la SEOM con criterios preestablecidos por un PNT diseñado a tal efecto que garantice el valor y los méritos de los datos aportados por cada Servicio.
   6. No creo que sea necesario compartir los datos clínicos de mis pacientes.
